# Does Parent Report Gross Motor Function Level of Cerebral Palsy Children Impact on the Quality of Life in these Children?

**Published:** 2017

**Authors:** Marzieh PASHMDARFARD, Malek AMINI, Reza shervin BADV, Narges Ghaffarzade NAMAZI, Mehdi RASSAFIANI

**Affiliations:** 1 Department of Occupational Therapy, Zanjan University of Medical Sciences, Zanjan, Iran.; 2 Department of Occupational Therapy, School of Rehabilitation Sciences, Iran University of Medical Sciences, Tehran, Iran.; 3 Children's Medical Center of Excellence, Tehran University of Medical Sciences, Tehran, Iran.; 4 Department of Occupational Therapy, Faculty of Allied Health, Kuwait University, Kuwait, Pediatric Neurorehabilittion Research Center, The University of Social Welfare and Rehabilitation Sciences, Tehran, Iran.

**Keywords:** Gross motor function, Quality of life, Cerebral palsy

## Abstract

**Objective:**

The aim of this study was to assess the effect of parent report gross motor function level of cerebral palsy (CP) children on the parent report quality of life of CP children.

**Materials & Methods:**

Sampling of this cross-sectional study was done in occupational therapy clinics and CP children’s schools in 2016 in Zanjan, Iran. Samples size was 60 CP children aged 6-12 yr and for sampling method, a non-probability convenience was used. For assessing the quality of life of CP children the cerebral palsy quality of life (CP QOL) questionnaire and for assessing the level of gross motor function of CP children the Gross Motor Function Classification System Family Report Questionnaire (GMFCSFRQ) were used.

**Results:**

The average age of children (22 males and 30 females) was 8.92 yr old (minimum 6 yr and maximum 12 yr). The relationship between the level of gross motor function and participation and physical health was direct and significant (r=0.65). The relationship between functioning, access to services and family health with the level of gross motor function was direct but was not significant (*P*>0.05) and the relationship between pain and impact of disability and emotional well-being with the level of gross motor function was significant (*P*<0.05).

**Conclusion:**

There was no strong correlation between the level of gross motor function and quality of life of children with cerebral palsy. It means that the level of gross motor function cannot be used as a predictor of quality of life for children with cerebral palsy alone.

## Introduction

Cerebral palsy (CP) is one of the most common childhood disorders that occur in the growing incidence of lesions in the brain (1). The prevalence of CP is approximately 2-2.5 people per 1000 live births (1). It begins early in life, but all affect the scope of one's life (1). Children with CP may experience wide range of disorders such as postural disorders, coordination disorders, sensory and intellectual disorders in their lifetime. Generally, this kind of cognitive and motor impairments which include those factors are most reasons that lead to limiting the children with CP in participating in all life areas (2, 3). Most people with CP are facing with limits on walking and other physical activities. The general idea is that the experience of such restrictions affects people's quality of life (2). Children with CP due to movement restrictions in terms of mobility and self-care are dependent on others and these restrictions affect their participation in activities of daily living (ADL) and consequently affect their quality of life (4-6). 

World Health Organization(WHO) defines quality of life as one's perception of his/her own position in relation to the objectives, expectations, values, and concerns, in the cultural and social context in his/her life. Quality of life is a multidimensional structure and is an overall assessment of well-being (2, 7). The United Cerebral Palsy Association (1991) adopted the mission statement: “to affect positively the quality of life of CP children’’ QOL has become the most important outcome of treatments and interventions for children with CP (8). 

The main goal of rehabilitation interventions, especially occupational therapy is to improve the quality of life and participation of children with CP in different aspects of life areas (9). Since the main problems of children with CP are disorders such as walking and manipulation, and particularly more focus of rehabilitation interventions and occupational therapy is on motor components such as strength and flexibility of muscles and joints, the question arises that, is focusing on promoting skills and motor components of children with CP ultimately leads to improvement of their quality of life or not? Therefore, this study aimed to determine the relationship between the level of Gross Motor Function of children with CP and their level of quality of life. 

## Materials & Methods:

 The sample size in this study was 60 parents of children with CP. In this cross-sectional study, the sampling was done in occupational therapy clinics and exceptional children’s schools in 2016 in Zanjan, Iran. 

The project proposal legislated with the ethics code ‘ZUMS.REC.1395.75’ in Zanjan University of Medical Sciences. Then an introduction letter from Zanjan University of Medical Sciences was taken and referred to occupational therapy clinics and exceptional children’s schools for sampling and getting information of them. 

In this study, a non-probability convenience sampling method was used. This means that after attending the clinics and schools, children who had inclusion criteria of this study were enrolled (Table 1).

**Table 1:** Inclusion and exclusion criteria

**Inclusion criteria**	**Exclusion criteria**
Age range of children be 6-12 yr old	Not wanting to continue collaborating in the study
Children diagnosed by a neurologist or pediatric neurologist to be a cerebral palsy patient	Failure to complete the full questionnaire
Not Having epilepsy or seizure disorder that is resistant to treatment	Filling the questionnaire by others instead of the parents or caregivers of CP children
Lack of other disorders such as progressive neuromuscular disorders	
Not having Orthopedic surgical pathology and Botox injections in the past 12 months	
Lack of blindness and deafness	
Having the IQ over than 70%	

First of all the consent forms were taken from the parents of children with CP and after those two questionnaires: (Cerebral Palsy Quality of Life (CP QOL) and GMFCS Family Report Questionnaire) were completed by CP children parents’ randomly. The two questionnaires were given to 60 parents of children with CP that had inclusions criteria, but because the 8 of these samples did not complete the questionnaire, were excluded from study and finally, 52 samples remained for final analysis. 

***Outcome measures***

CP QOL: In order to collect information about the quality of life of children with CP, CP QOL questionnaire was used. Validity and reliability of this questionnaire in Iran were assessed, that test-retest reliability for the subscales has been reported 0.74- 0.84(10). CP QOL Questionnaire has 65 items that assesses the CP children’s quality of life in 7 categories: (Social well-being and acceptance (12 items), Functioning (12 items), Participation and physical health (11 items), Emotional well-being (6 items), Access to services (12 items), Pain and impact of disability (8 items), Family health (4 items))(10).

GMFCS Family Report Questionnaire: In order to collect information about the level of gross motor function of children with CP, GMFCS Family Report Questionnaire was used (11). Validity and reliability of this questionnaire in Iran were assessed, and the Intraclass Correlation Coefficient (ICC=0.92) for the total score of the questionnaire was reported (12). GMFCS Family Report Questionnaire is an observational standard classification system that categorizes children with CP in 5 levels based on the current gross motor abilities, limitations in gross motor functions and the need for technology and assistive devices (11).

***Data analysis method***

For data analysis, the SPSS ver. 21 (Chicago, IL, USA) was used. To determine the normal distribution of data, the Kolmogorov-Smirnov test (K-S) and to measure the relationship between variables, Spearman correlation test was used.

## Results

***Descriptive Statistics***

The mean age of children was 8.92 yr (min: 6 and max: 12 yr), and 22 of the children were male and 30 were female (Table 2).

**Table 2:** Democratic characteristic of samples

		**Frequency**	**Percent (100%)**
**Age (yr)**	6 yr old	5	9.6
	7 yr old	9	17.3
	8 yr old	9	17.3
	9 yr old	9	17.3
	10 yr old	8	15.4
	11 yr old	6	11.5
	12 yr old	6	11.5
**Gross Motor Function Levels** **(GMFCS** **levels) **	I	12	23.1
	II	2	3.8
	III	6	11.5
	IV	15	28.8
	V	17	32.7
**Gender**	Male	22	42.3
	Female	30	57.7
**Type of Cerebral Palsy**	Hemiplegia	14	26.9
	Diplegia	25	48.1
	Quadriplegia	13	25.0

***Statistics analysis***

The data distribution was not normal (*P*<0.05). To analyze the relationship between variables the Spearman test was used. The results of analysis of the relationship between the level of gross motor function and quality of life of children with CP are given in Table 3. The relationship between participation and physical health with the level of gross motor function was direct and significant (*r*=0.65). The relationship between functioning, access to services and family health with the level of gross motor function were direct but were not significant (*P*>0.05). The relationship between pain and impact of disability and emotional well-being with the level of gross motor function was significant (*P*<0.05) (Table 3).

**Table 3:** Correlation between the Domains of CP- QOL, and GMFCS

Domains of CP QOL-Child	*P*	r^2 ^(%)	r	SD	Mean	n
Social well-being and acceptance	0.03	7.8	0.28**	21.09	101.57	52
Functioning	0.21	2.5	0.16	8.45	32.94	52
Participation and physical health	0.00	42	0.65**	22.39	91.26	52
Emotional well being	0.002	16	-0.41**	6.18	4.48	52
Pain and impact of disability	0.01	10	-0.33**	14.66	41.55	52
Access to service	0.41	1.4	0.12	15.49	41.90	52
Family health	0.71	0.16	0.04	8.78	28.09	52

** *P<0.05*

## Discussion

The aim of this study was to examine the relationship between the level of gross motor function and quality of life of children with CP in Zanjan, Iran. The quality of life of children with CP is lower than to healthy peers (12). The best way to assess the quality of life of children with CP is using a questionnaire that is able to measure the quality of life of children with CP in all physical, psychological, social, and other aspects of their life (13). For achieving more accuracy, the parent report version of CP QOL-Child questionnaire was used. The results of this study showed that the relationship between social well-being and acceptance item of quality of life with gross motor function level of CP children was weak; this result is similar to another study (14). 

Social well-being and acceptance item of quality of life may be influenced by the factors such as culture, economic level, and social context. In this study, the relationship between the participation and physical health with the level of gross motor function was direct and significant that is consistent with the study in Hamedan, Iran (14). According to the results, in the procedure of rehabilitation, if the therapists pay attention to promoting the gross motor functions and skills of CP children, their participation skills, and their physical well-being will improve, especially the skills that need more mobility. The items of participation and physical health consist the activities that require movement and mobility. The significant correlation of participation and physical health with the level of gross motor function is unexpected. Nevertheless, the relationship between functioning, access to services and family health with the level of gross motor function were direct but were not significant and the relationship between them is so weak. 

The CP children’s quality of life is a multidimensional concept influenced by many factors such as physical status, environmental issues and context and factors such as the level of gross motor function and the level of spasticity have much less impact on quality of life (15). Therefore, occupational therapists should not pay attention only to the Gross motor function of children with CP, and they have to pay attention to other concepts of their quality of life by environmental adaptations, educating the parents and caregivers etc. therapists by conducting a comprehensive and multidimensional intervention can promote the CP children’s quality of life. However, the improvement in gross motor skills of children with CP affects improving the quality of life in all aspects of the physical and psychological life (5). The quality of life of children with CP and their parents are lower than their healthy peers are, but the factors that affect the CP children’s quality of life and the factors that affect their parent’s quality of life are different. 

The main factors that affect the children with CP quality of life are as follows: Low levels of health and socioeconomic levels compared to healthy peers. In addition, the main factors that affect their parents quality of life are difficulty in accessing to rehabilitation services, low level of socio economic status, and their children’s disability, but there is not any relationship between the severity of disability and their parent’s quality of life (16). The quality of life of children with CP in all aspect of life areas (physical and psychosocial) are similar to their healthy peers but the level of their parent’s quality of life is lower than other parents, and the probable reason was because of the low level of gross motor function of children with CP and their disability compared with healthy children (17). Different results may be because of cultural and attitudinal factors that govern the society. The relationship between pain and impact of disability with the level of gross motor function was significant but diverse; likely whatever the level of gross motor function of children with CP is high the level of pain and the impact of pain on disability are low. The result of this study is aligned with other that indicated the main reason for the low quality of life of children with CP is pain (18). The significant relationship between pain and gross motor function of children with CP may be because, when the level of gross motor function is low and the severity of spasticity is high then the mobility of the body will decrease and the probability of deformity and contracture rises out and these factors can increase the feeling of pain. Using GMFCS and the level of gross motor function in children with CP were good predictor of quality of life of the CP children's parents, not the children with CP (19). 

**In Conclusion, **there is not a strong correlation between level of gross motor function and quality of life of children with CP (20). It should be considered on the treatment of these children that the level of gross motor function could not be alone a predictor of quality of life of children with CP. In the rehabilitation interventions of children with CP, occupational therapists should not focus only on gross motor function of children with CP but also for promoting the quality of life of these children, they have to pay attention to the cultural, environmental, economic, social and physical condition of children with CP and their parents and caregivers. 

## Conflict of Interest

The authors declare no potential conflicts of interest with respect to the research, authorship, and/or publication of this article.
